# Potent antitumor activity of a bispecific T-cell engager antibody targeting the intracellular antigen KRAS G12V

**DOI:** 10.17305/bb.2024.10431

**Published:** 2024-10-01

**Authors:** Changchang Lu, Lu Zou, Qiaoli Wang, Mengna Sun, Tianyu Shi, Shuang Xu, Fanyan Meng, Juan Du

**Affiliations:** 1 Department of Oncology, Nanjing Drum Tower Hospital, Clinical College of Nanjing Drum Tower Hospital, Nanjing University of Chinese Medicine, Nanjing, China; 2 Institute of Translational Medicine, Zhejiang University, Hangzhou, China; 3 The Comprehensive Cancer Center of Nanjing Drum Tower Hospital, Affiliated Hospital of Medical School, Nanjing University, Nanjing, China; 4 Department of Laboratory Medicine, Nanjing Drum Tower Hospital, Clinical College of Nanjing Medical University, Nanjing, China

**Keywords:** Bispecific antibody, Kirsten Rat Sarcoma viral oncogene homolog (KRAS) G12V, intracellular tumor antigen, cancer

## Abstract

Kirsten Rat Sarcoma viral oncogene homolog (KRAS) is one of the most frequent oncogenes. However, there are limited treatment options due to its intracellular expression. To address this, we developed a novel bispecific T-cell engager (BiTE) antibody targeting HLA-A2/KRAS G12V complex and CD3 (HLA-G12V/CD3 BiTE). We examined its specific binding to tumor cells and T cells, as well as its anti-tumor effects in vivo. HLA-G12V/CD3 BiTE was expressed in *Escherichia coli* and its binding affinities to CD3 and HLA-A2/KRAS G12V were measured by flow cytometry, along with T-cell activation. In a xenograft pancreatic tumor model, the HLA-G12V/CD3 BiTE’s anti-tumor effects were assessed through tumor growth, survival time, and safety. Our results demonstrated specific binding of HLA-G12V/CD3 BiTE to tumor cells with an HLA-A2/KRAS G12V mutation and T cells. The HLA-G12V/CD3 BiTE also activated T-cells in the presence of tumor cells in vitro. HLA-G12V/CD3 BiTE in vivo testing showed delayed tumor growth without severe toxicity to major organs and prolonged mouse survival. This study highlights the potential of constructing BiTEs recognizing an HLA-peptide complex and providing a novel therapy for cancer treatment targeting the intracellular tumor antigen.

## Introduction

Recently, cancer immunotherapy has become one of the main forces of treatment for tumors. In particular, immune checkpoint inhibitors, such as programmed cell death protein-1 (PD-1) and cytotoxic T lymphocyte-associated protein-4 (CTLA-4) blockade are the focus of tumor therapy [1], which have shown outstanding efficacy in non-small cell lung cancer (NSCLC) [2], gastric cancer [3], and a series of clinical trials in different tumors are ongoing.

An optimal cancer therapy is premised on the idea that tumor cells can be accurately identified and killed without damaging normal cells. Therefore, the key to designing a promising therapy is to find a suitable tumor antigen. Tumor antigens are mainly divided into intracellular and extracellular antigens [4]. Tumor-specific antigens (TSAs) are ideal targets for many cancer immunotherapies while most of them are intracellular proteins and thus not accessible to many popular therapies including chimeric antigen receptor T-cell immunotherapy (CAR-T) and bispecific antibody [5–7]. Using a major histocompatibility complex (MHC) to present antigenic peptides to the cell surface is a promising way to overcome the limitation of intracellular protein [8]. There is a large existing community using peptide-MHC (pMHC) to specifically target intracellular antigens. T cell receptor-engineered T cell (TCR-T) therapy is the main treatment using pMHC to target tumors and shows remarkable efficacy [9, 10]. Also, many antibodies targeting peptide-human leukocyte antigen complexes have been designed for cancer therapy, especially using the MAGE-HLA complex [11–13].

Bispecific T cell engager (BiTE) therapy is considered a potential immunotherapy that binds to a selected tumor association antigen (TAA) and CD3 molecules [14, 15]. Blinatumomab, a CD19/CD3 BiTE, is the first BiTE approved by the U.S. Food and Drug Administration for the treatment of Philadelphia chromosome-negative relapsed or refractory B-cell precursor acute lymphoblastic leukemia [16]. Recently, different types of BiTE have emerged continuously, including targeting EpCAM [17], EGFRvIII [18], CEA [19], and so on. However, all of them are in clinical or preclinical stages. And there is no BiTE targeting popular TSA such as KRAS because of its intracellular mutation [4, 20]. Single-chain variable fragments (scFv) for KRAS-HLA complexes have been generated [21] and there is a study converting these scFvs to the single-chain diabody which can induce T cell activation and kill target cancer cells [22].

Herein, we generated a novel BiTE targeting HLA-A2/KRAS G12V complex and characterized the in vitro features, including binding to target cells and promoting T-cell activation. We also evaluated the anti-tumor effect in in vivo of HLA-G12V/CD3 BiTE in xenograft models. Our goal was to explore a new strategy for the BiTE format to be applied to a much larger universe of targets.

## Materials and methods

### Generation, expression, and purification of HLA-G12V/CD3 BiTE

HLA-G12V/CD3 BiTE was designed in the following orientation: (anti-HLA-A2-KRAS G12V scFv) - (anti-CD3 scFv) - His tag. The anti-HLA-A2-KRAS G12V scFv was developed as previously described [21] and we chose the D10-7 scFv sequence. The anti-CD3 scFv was derived from an antibody specific for CD3ɛ on T cells. DNA fragments were synthesized and cloned into bacterial expression vector pET28a by iCarTab (Suzhou, China). The recombinant plasmid was confirmed through DNA sequencing by Sangon Biotech (Shanghai, China). The plasmid was transfected into *Escherichia coli* BL21 (DE3) and induced by isopropyl β-D-1-thiogalactopyranoside (IPTG). The bacteria were disrupted using a supersonic technique. The inclusion bodies were washed and dialyzed in a concentration gradient of urea (8M-6M-4M-3M-2M-1M urea) at 4 ^∘^C every 12 h with rapid mixing, followed by dialysis with only phosphate-buffered saline (PBS) for 24 h at 4 ^∘^C. The production was subjected to western blot (WB) analysis using an anti-His antibody (Abcam, ab5000) for confirmation. The HLA-G12V/CD3 BiTE was purified by HisTrap HP column (GE Healthcare, CT, USA) through the SDA-100 system (CELLPRO, Suzhou, China). The eluted protein was verified by 12% sodium dodecyl sulfate-polyacrylamide gel electrophoresis (SDS-PAGE) analysis. The protein was then quantitated with the BCA assay kit (NCM Biotech, Suzhou, China), filtered through a 0.22 µm sterile filter, and stored at −20 ^∘^C for future use.

### Cell lines and cell culture

The Cell Bank of Shanghai Institute of Biochemistry and Cell Biology provided the following human cancer cell lines: CFPAC-1, Capan-1, and Panc0504 for pancreatic cancer, NUGC-4 for gastric cancer, and SW480 for colon cancer. All the pancreatic cancer cell lines were cultured in Dulbecco’s Modified Eagle Medium (DMEM) medium containing 10% fetal bovine serum (FBS) at 37 ^∘^C and 5% CO_2_. NUGC-4 and SW480 were cultured in Roswell Park Memorial Institute (RPMI) 1640 medium containing 10% FBS at 37 ^∘^C and 5% CO_2_.

### Peripheral blood mononuclear cells (PBMCs) isolation and activation of human T cells

Peripheral blood mononuclear cells (PBMCs) were obtained from healthy donor blood and isolated using Ficoll density gradient centrifugation. PBMCs were cultured in AIM-V medium (Gibco, USA) for 2–4 h for adherence. Suspended T lymphocytes were harvested and expanded in AIM-V medium containing 10% FBS, 300 U/mL IL-2 (Peprotech, USA), 50 ng/mL IL-7 (PeproTech, USA), and 50 ng/mL IL-15 (PeproTech, USA). On day 2, the T cells were activated by adding 50 ng/mL OKT3 (eBioscience, USA) to the medium.

### Binding analysis of HLA-G12V/CD3 BiTE

Tumor cells were washed by PBS and then digested with trypsin. Tumor cells were resuspended in DMEM or RPMI1640 medium containing 10% FBS at 5×10^5^/mL and 200 µL of the cell suspension was incubated with HLA-G12V/CD3 BiTE for 60 min at room temperature. Subsequently, cells were washed with PBS twice and incubated with anti-His tag antibody at 4 ^∘^C for 40 min. T cells were resuspended in AIM-V medium at 5×10^5^/mL. Similar to the tumor cells, 200 µL of this T cell suspension was incubated with varying doses of HLA-G12V/ CD3 BiTE for 60 min at room temperature. After incubation, the T cells were also washed and stained with anti-His tag antibody for 40 min at 4 ^∘^C. Finally, flow cytometry analysis was conducted on all samples using Epics XL (Beckman Coulter, Brea, CA, USA) and the data was analyzed using FlowJo V.10.4 software.

### Activation of T lymphocytes and cytotoxicity assays

To examine T cell activation, fresh total T cells, T cells cocultured with KRAS G12V mutation, and KRAS WT tumor cells at an effector-to-target (E:T) ratio of 10:1 with or without HLA-G12V/CD3 BiTE (final concentration 10 µg/mL) were incubated in 96-well plate for 24 h at 37 ^∘^C. The supernatant fluids after 24 h were harvested for the secretion of interferon-γ (IFN-γ) using the BD CBA Human IFN-γ Flex Set (BD Bioscience, USA). Furthermore, we detected the IFN-γ release under different concentrations of HLA-G12V/CD3 BiTE. T-cell activation was also assessed using flow cytometry with the following antibodies: anti-CD3-PE (Beckman Coulter, USA), anti-CD4-APC (Beckman Coulter, USA), anti-CD8-FITC (RPA-T8, BD Bioscience), anti-CD69-PC5 (Beckman Coulter, USA), and anti-CD25-APC (Beckman Coulter, USA).

For cytotoxicity assays, CFPAC-1 cells were initially marked with Carboxyfluorescein succinimidyl ester (CFSE) (Invitrogen, USA). Then PBMC was added into the tumor cells at an effector-to-target ratio (E:T) of 10:1 with different concentrations of HLA-G12V/CD3 BiTE. The mixed cells were left to incubate for 48 h at 37 ^∘^C and then treated with Propidium iodide (PI) (Sigma, USA) for 10 min in the absence of light. Subsequently, the cells were washed and examined using flow cytometry.

### In vivo experiment

In order to evaluate the targeting capabilities and effectiveness against tumors, a humanized pancreatic cancer model (CFPAC-1) was utilized in vivo. An equal mixture of 2×10^6^ CFPAC-1 cells and Matrigel (BD Biosciences) was injected subcutaneously into the right flank of female BALB/c nude mice aged 6–8 weeks. For in vivo fluorescence imaging, HLA-G12V/CD3 BiTE was labeled with Cy5-NHS (APExBIO, USA) and injected intravenously into CFPAC-1 tumor-bearing mice. For resected tissue imaging, the mice were sacrificed at 3, 7, 24, and 48 h after injection. Tumors and main organs, including liver, lung, and kidney were excised and imaged.

**Figure 1.**
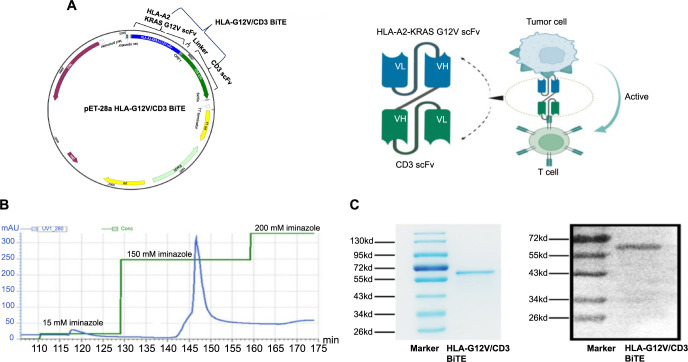
**Generation of HLA-G12V/CD3 BiTE**. (A) The schematic map of plasmid expressing HLA-G12V/CD3 BiTE and the mechanism of HLA-G12V/CD3 BiTE. Linker, (GGGGS)3; (B) Purification profile of HLA-G12V/CD3 BiTE; (C) Eluted fractions are identified by SDS-PAGE (left) and western blot (right). HLA-G12V/CD3 BiTE: Bispecific T-cell engager antibody targeting HLA-A2/KRAS G12V complex and CD3; SDS-PAGE: Sodium dodecyl sulfate-polyacrylamide gel electrophoresis.

Once the tumors reached a size of 80–100 mm^3^, five groups of mice with pancreatic tumors were randomly assigned to receive one of the four treatments (*n* ═ 5/group): PBS (control); HLA-G12V/CD3 BiTE (100 µg, day 1–5); PBMC (1×10^7^, day 1 and day 5); PBMC+BiTE (PBMC, 1×10^7^, day 1 and day 5; BiTE, 100 µg, day 1–5). T cells and all the reagents were administered via the tail vein. Tumor sizes and weight were measured every other day. Tumor volume was calculated using the formula length×width^2^×0.5. When the mice reached ethical human endpoints, such as a tumor volume of 1500 mm^3^, tumor ulceration, or a decline in health, they were euthanized.

To evaluate T-cell infiltration in tumors, mice were euthanized after receiving either PBMC or PBMC+BiTE treatment. Subcutaneous tumors were separated, minced, and homogenized into single-cell suspensions. Cells were washed with sterile PBS and stained with hCD3 mAb (Beckman Coulter, USA) for flow cytometric analysis.

To evaluate the safety of HLA-G12V/CD3 BiTE in vivo, major organs were harvested, fixed in 4% paraformaldehyde, sectioned, and stained with the HE staining technique.

### Ethical statement

All animal procedures were carried out following the guidelines set by the Animal Care Committee at Drum Tower Hospital (Nanjing, China) and the Ethics Committee of Drum Tower Hospital approved all experiments in this study.

### Statistical analysis

All data were analyzed and graphed by SPSS and GraphPad Prism V.9.2.0 (GraphPad Software, San Diego, CA, USA). Data are presented as means ± SEM. Statistical significance between different experimental groups was analyzed by student’s *t* test or two-way ANOVA. A *P* value of less than 0.05 was considered significant.

## Results

### Generation and characterization of HLA-G12V/CD3 BiTE

**Figure S1.**
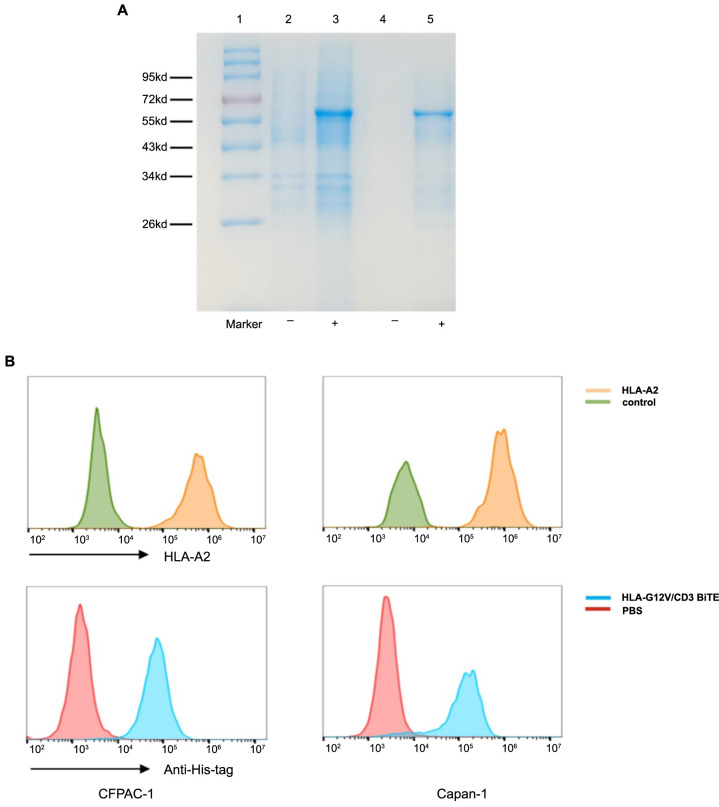
(A) HLA-G12V/CD3 BiTE is induced and expressed as inclusion body. Lane 1, marker; lane 2, bacterial cultures without IPTG, lane3, bacterial cultures with IPTG; lane 4, inclusion bodies without IPTG; lane 5, inclusion bodies with IPTG; (B) The comparison of HLA-A2 expression (above) and the binding to HLA-G12V/CD3 BiTE (below) of CFPAC-1 (left) and Capan-1 cells (right). The results are representative of at least three independent experiments. HLA-G12V/CD3 BiTE: Bispecific T-cell engager antibody targeting HLA-A2/KRAS G12V complex and CD3.

HLA-G12V/CD3 BiTE was generated by starting with protein sequences of scFv specific for HLA-A2/KRAS G12V complex at the N-terminus, interacting with scFv of the anti-CD3 antibody at the C-terminus through a flexible 15-amino-acid (GGGGS)_3_ linker. To enable detection and purification, a His tag was added to the 3’-end of the CD3 VL fragment (Figure 1A). The fusion protein was successfully induced in *E. coli* BL21 and mainly expressed as an inclusion body with an approximate size of 57 kDa in SDS-PAGE (Figure S1A). Refolded fusion protein was purified by using a Ni-NTA column and the target protein was eluted when the concentration of iminazole reached 150 mM, which showed one spiculate and symmetrical elute peak in the linear elution profiles (Figure 1B). Purity was confirmed by 12% SDS-PAGE and the protein’s molecular size was verified through WB analysis (Figure 1C).

### Binding capacity of HLA-G12V/CD3 BiTE to KRAS G12V cells

**Figure 2.**
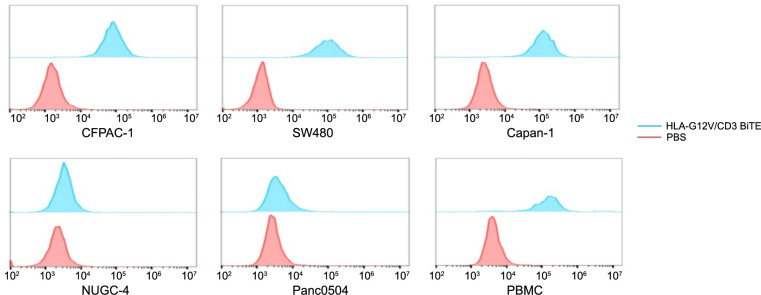
**Flow cytometry results show bindings of HLA-G12V/CD3 BiTE to tumor cells with different KRAS mutation types and T cells**. The results are representative of at least three independent experiments. HLA-G12V/CD3 BiTE: Bispecific T-cell engager antibody targeting HLA-A2/KRAS G12V complex and CD3; KRAS: Kirsten Rat Sarcoma viral oncogene homolog; PBS: Phosphate-buffered saline.

To examine the in vitro binding of HLA-G12V/CD3 BiTE, we chose five kinds of tumor cells with different KRAS mutations. We used flow cytometry to determine the binding activities of HLA-G12V/CD3 BiTE to tumor cells (Figure 2). Results showed specific binding of HLA-G12V/CD3 BiTE to CFPAC-1, Capan-1, and SW480 cells, all of which have the KRAS G12V mutation. However, HLA-G12V/CD3 BiTE showed no binding capacity to NUGC-4 (KRAS WT) and Panc0504 cells (KRAS G12D). HLA-A2 expression was also measured on CFPAC-1 and Capan-1 tumor cells using the HLA-A2 monoclonal antibody (BB7.2, eBioscience, USA). The high HLA-A2 expression of tumor cells was consistent with their high binding rate to HLA-G12V/CD3 BiTE (Figure S1B).

### HLA-G12V/CD3 induced tumor-specific T-cell activation and promoted tumor lysis

**Figure 3.**
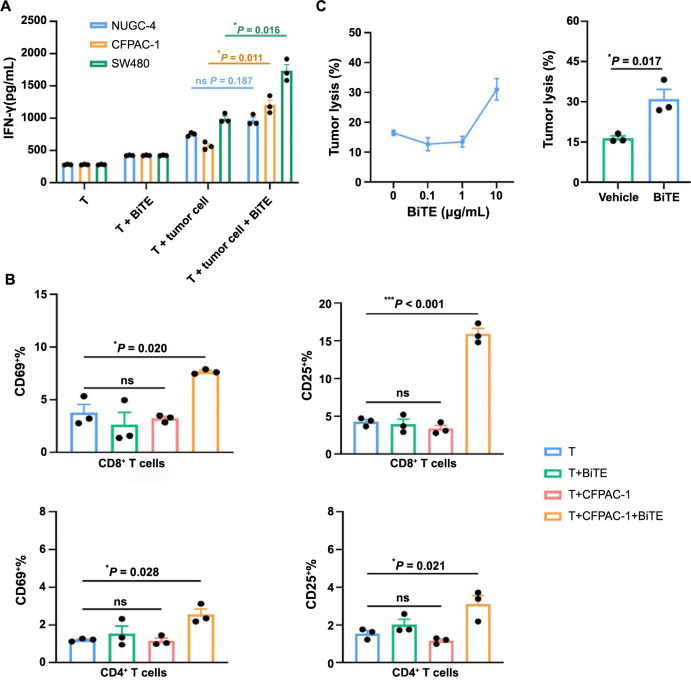
**Flow cytometry results show the activation of T cells and cytotoxicity of HLA-G12V/CD3 BiTE**. (A) The secretion of IFN-γ increases in the co-culture of target tumor cells and HLA-G12V/CD3 BiTE; (B) HLA-G12V/CD3 BiTE stimulates T cells and the expression of CD69 and CD25 both increase after incubation with CFPAC-1 tumor cells; (C) The dose-response of tumor lysis of HLA-G12V/CD3 BiTE at an E:T ratio of 10:1. The results are representative of at least three independent experiments. *P* value: *P < 0.05, **P < 0.01, ***P < 0.001. ns: Not significant; HLA-G12V/CD3 BiTE: Bispecific T-cell engager antibody targeting HLA-A2/KRAS G12V complex and CD3.

**Figure S2.**
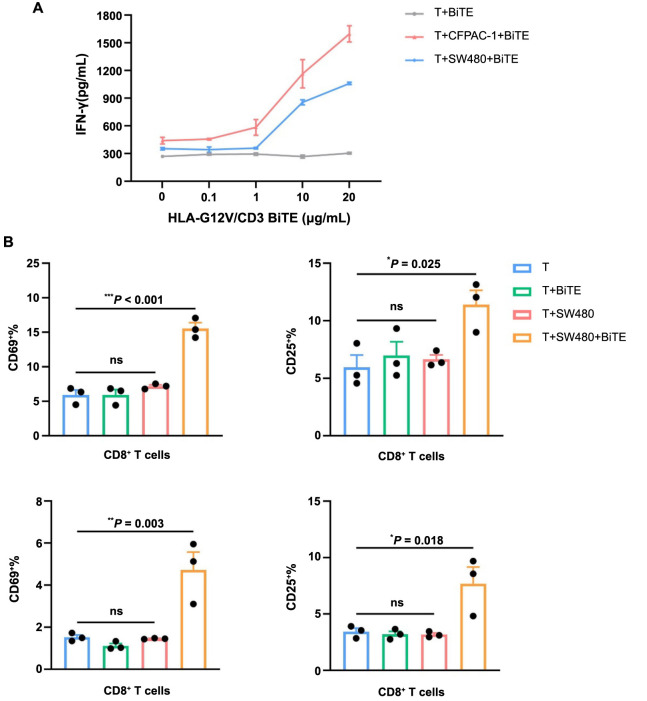
(A) The dose-response of IFN-γ secretion in the presence of target tumor cells; (B) HLA-G12V/CD3 BiTE stimulates T cells and the expression of CD69 and CD25 both increase after incubation with SW480 tumor cells. The results are representative of at least three independent experiments. *P* value: *P < 0.05, **P < 0.01, ***P < 0.001. ns: Not significant; HLA-G12V/CD3 BiTE: Bispecific T-cell engager antibody targeting HLA-A2/KRAS G12V complex and CD3; IFN-γ: Interferon-γ.

We next investigated the ability of HLA-G12V/CD3 BiTE to bind to T cells and activate T cells. We isolated PBMCs and the results of flow cytometry showed that HLA-G12V/CD3 BiTE displayed positive binding to T cells (Figure 2). We then compared the expression of IFN-γ in T cells activated by HLA-G12V/CD3 BiTE. The results of the production of IFN-γ showed that HLA-G12V/CD3 BiTE can only significantly activate T cells in the presence of KRAS G12V mutation tumor cells (Figure 3A). Moreover, the release of IFN-γ was found to increase with increasing concentrations of HLA-G12V/CD3 BiTE in the presence of target tumor cells (Figure S2A). In addition, surface expression of T cell activation markers CD69 and CD25 elevated in both CD8^+^ and CD4^+^ T cells in the presence of CFPAC-1 tumor cells and HLA-G12V/CD3 BiTE (Figure 3B). Same results were obtained on co-culture of HLA-G12V/CD3 BiTE and SW480 tumor cells (Figure S2B). The results suggested that HLA-G12V/CD3 BiTE can stimulate tumor-specific T-cell activation in the presence of target cells.

We then evaluated the cytotoxicity of HLA-G12V/CD3 BiTE. A dose-dependent cytotoxicity effect caused by HLA-G12V/CD3 BiTE was observed at an E:T ratio of 10:1. At this ratio, the presence of HLA-G12V/CD3 BiTE resulted in a 30% killing rate, while the control group only had a 16% (Figure 3C).

### HLA-G12V/CD3 targeted tumors and promoted T-cell infiltration in mouse tumors

**Figure 4.**
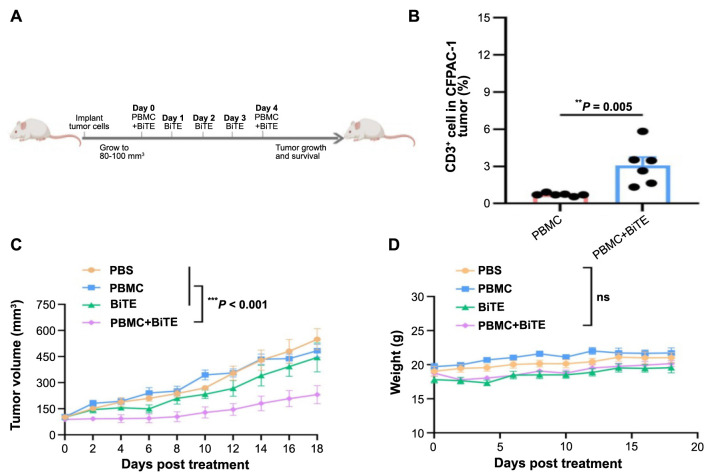
**HLA-G12V/CD3 BiTE inhibits in vivo tumor growth and improves mouse survival (*n* ═ 5/group) in CFPAC-1 subcutaneous mouse model**. (A) Treatment schema of HLA-G12V/CD3 BiTE; (B) HLA-G12V/CD3 BiTE increases the infiltration of T-cells into tumor tissue in CFPAC-1 subcutaneous mouse model. Tumor growth profiles (C) and weight profiles (D) in CFPAC-1 subcutaneous mouse model. *P* value: *P < 0.05, **P < 0.01, ***P < 0.001. ns: Not significant; HLA-G12V/CD3 BiTE: Bispecific T-cell engager antibody targeting HLA-A2/KRAS G12V complex and CD3; PBS: Phosphate-buffered saline; PBMC: Peripheral blood mononuclear cells.

**Figure S3.**
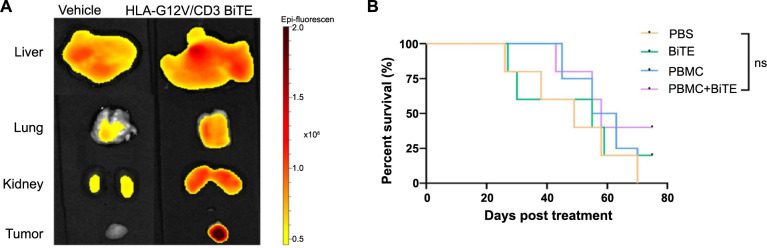
(A) Ex vivo images of tumors, livers, lungs and kidneys collected at 7 h after injection; (B) Survival analysis in CFPAC-1 subcutaneous mouse model. *P* value: ns: Not significant.

We assessed HLA-G12V/CD3 binding to KRAS G12V mutation tumor. Experimental animals were injected intravenously with a Cy5-NHS labeled HLA-G12V/CD3 BiTE and fluorescence reflectance imaging at 7 h revealed a stronger signal in the tumor compared to other organs (Figure S3A). This confirms the specific targeting of the KRAS G12V mutation tumor in vivo by HLA-G12V/CD3 BiTE. We then investigated whether HLA-G12V/CD3 BiTE could promote T-cell infiltration in mouse tumors. Flow cytometry analyses of T-cell infiltration in tumors showed that HLA-G12V/CD3 BiTE significantly increased the percentage of human T-cells (CD3+ population) in tumors in comparison to the control groups (Figure 4B).

### In vivo anti-tumor efficacy of HLA-G12V/CD3 BiTE

To evaluate the effects of HLA-G12V/CD3 BiTE on tumor growth, we established a pancreatic cancer xenograft tumor model in nude mice using CFPAC-1. After the tumor volume reached around 80–100 mm^3^, the mice were treated with HLA-G12V/CD3 BiTE (Figure 4A). We first evaluated anti-tumor effects in mice bearing pancreatic tumors. By following tumor growth, we found that the administration of 100 µg HLA-G12V/CD3 BiTE per day for five days and injection of PBMC on day 1 and day 5 significantly delayed tumor growth. However, injections of PBMC or HLA-G12V/CD3 BiTE alone did not significantly differ in tumor growth compared to the control group receiving PBS (Figure 4C). HLA-G12V/CD3 BiTE did not affect mouse weights (Figure 4D). While the survival times varied among the different groups, the group receiving HLA-G12V/CD3 BiTE + PBMC had a longer survival time, though without statistical significance (Figure S3B).

### Toxicology study of HLA-G12V/CD3 BiTE in major organs

**Figure 5.**
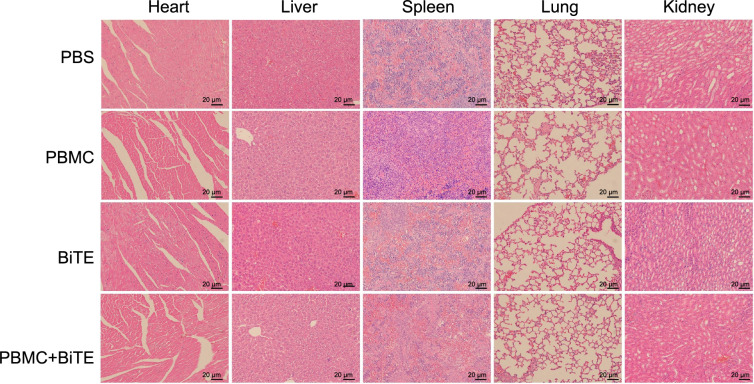
**The in vivo toxicity of HLA-G12V/CD3 BiTE.** HLA-G12V/CD3 BiTE: Bispecific T-cell engager antibody targeting HLA-A2/KRAS G12V complex and CD3; PBS: Phosphate-buffered saline; PBMC: Peripheral blood mononuclear cells.

To assess the in vivo safety of HLA-G12V/CD3 BiTE, we conducted HE staining on major organs of different groups, including the heart, liver, spleen, lungs, and kidneys (Figure 5). Our results show no signs of severe toxicity. Furthermore, there was no evidence of lymph node infiltration or tumor metastasis, indicating the safety and efficacy of HLA-G12V/CD3 BiTE.

## Discussion

In this study, we generated HLA-G12V/CD3 BiTE, a bispecific antibody that can specifically target T cells and HLA-A2 positive tumor cells with KRAS mutation simultaneously. HLA-G12V/CD3 BiTE engagers T cells and target tumor cell and promotes the formation of an immunological synapse. T cells are activated and begin to proliferate and then tumor cell lysis is triggered. In vitro results showed increased IFN-γ secretion, CD69, and CD25 expression upon co-culturing with HLA-G12V/CD3 BiTE and tumor cells. In vivo, BiTE directs T cells to the tumor site through the bloodstream. However, the dense tumor microenvironment may limit T cell recruitment and infiltration, resulting in suboptimal anti-tumor effects. This study is a novel approach to targeting the peptide-HLA complex with BiTE in solid tumors. Data reveal the feasibility of overcoming limitations in intracellular antigen recognition by traditional tumor-specific monoclonal antibodies (mAbs). In vitro, HLA-G12V/CD3 BiTE binds to both tumor cells and primary T cells, resulting in inhibited tumor growth and prolonged mouse survival to some extent.

KRAS is frequently mutated in a variety of cancers, especially pancreatic cancer and colon cancer [23]. The most commonly mutated site for this gene is codon 12, which includes G12D (45%), G12V (35%), G12R (17%), and a few other uncommon mutations [24, 25]. Sotorasib, a small molecule that specifically inhibits KRAS G12C, is the first RAS GTPase family inhibitor approved for KRAS G12C-mutated NSCLC [26, 27]. Cancer immunotherapy has shown outstanding efficacy in most tumor therapies. However, no immunotherapies targeted KRAS mutation have been approved for clinical treatment although some bispecific antibodies targeting mutant RAS neoantigens have shown the ability to treat cancer in mouse models [22]. Some potential treatment therapies including chimeric antigen receptor (CAR) T cell therapy [28], T cell receptor (TCR)-engineered T cell therapy [29], and bispecific mAbs [30], which all have succeeded in clinical practice [31], while none of them targeted KRAS mutation because it is an intracellular mutant antigen.

Bispecific mAbs have shown potential anti-tumor efficacy in both preclinical and clinical studies. However, many identified tumor antigens are intracellular proteins that cannot be targeted by mAbs that only work against cell-surface targets [32, 33]. To overcome this limitation, some researchers have drawn inspiration from TCR mimic mAbs, which are able to target peptides presented on the cell surface in complex with HLA [34]. One study utilized a BiTE derived from the TCR mimic mAb ESK1, which binds specifically to Wilms’ tumor protein (WT1) in the context of HLA. This BiTE activates T cells and effectively kills tumor cells in vitro and in vivo, demonstrating its efficacy in both acute myeloid leukemia and solid tumors [35]. Tebentafusp, a bispecific targeting gp100-peptide-HLA has been approved to HLA-A*02:01-positive patients with metastatic uveal melanoma and the clinical data showed a proven overall survival benefit [36]. Similarly, we constructed HLA-G12V/CD3 BiTE on the basis of the anti-HLA-A2-KRAS G12V scFv selected as previously described [21]. Unlike many tumor-associated antigens (TAA), KRAS is classified as a TSA, meaning it is only expressed in tumor cells and does not cause on-target off-tumor side effects. Our results demonstrated that it was feasible for BiTE to specially target and kill tumor cells expressing peptide-HLA complex on the cell surface both in vivo *and* in vitro, providing evidence for expending the application scope of BiTE in intracellular tumor antigens.

In our study, we generated HLA-G12V/CD3 BiTE through *E. coli* BL21 (DE3) to preliminarily explore its anti-tumor effect because this expression system offers simplicity compared to eukaryotic systems. However, it often results in protein inclusion bodies, requiring denaturation, renaturation, and purification for an active refolded protein. In contrast, a eukaryotic vector can produce active proteins without denaturation. Using a yeast or mammalian expression system may be beneficial, but further testing on stability and activity under different systems is needed. While HLA-G12V/CD3 BiTE delayed tumor growth, there was no significant effect on survival time. This could be due to the BiTE’s short half-life and immunosuppression in pancreatic tumors, or the chosen therapeutic regimen. More therapies are necessary to determine the most effective treatment protocol. Moreover, the binding strength between HLA-G12V/CD3 BiTE and T cells is relatively weak, and the dense matrix surrounding tumors can limit T cell infiltration and hinder effective tumor elimination. To enhance anti-tumor efficacy, we plan to combine HLA-G12V/CD3 BiTE with immune checkpoint blockade or chemotherapy. One study showed that combining iRGD-anti-CD3 with PD-1 blockade improved anti-tumor efficacy and prolonged survival in mice with subcutaneous tumors [37]. Furthermore, ERY974, a type of BiTE that targets glypican-3, significantly increased T cell infiltration and induced tumor regression when combined with chemotherapy [38].

## Conclusion

In summary, we generated a novel design of BiTE targeting peptide-HLA complex and our data suggested that it was effective and safe both in vitro and in vivo. This approach broadens the targeting scope of tumor antigens, especially intracellular tumor antigens. We expect that this approach will open up possibilities for treating KRAS mutation tumors.

## Supplemental data

## Data Availability

The datasets used in this study are available from the corresponding author on reasonable request.
